# Imaging biomarkers in neurodegenerative diseases: advances and challenges

**DOI:** 10.3389/fnagi.2026.1813588

**Published:** 2026-06-26

**Authors:** Shreyasi Majumdar, Puneet K Samaiya, Sahabuddin Ahmed, Santosh Kumar Prajapati

**Affiliations:** 1 Department of Pharmaceutical Technology, School of Health and Medical Sciences, Adamas University, Kolkata, India; 2 Department of Pharmacy, Shri G. S. Institute of Technology and Science, Indore, India; 3 Department of Psychiatry, Yale University School of Medicine, New Haven, CT, United States; 4 Department of Neurosurgery, Brain and Spine, Center of Excellence in Aging and Brain Repair, University of South Florida Morsani College of Medicine, Tampa, FL, United States

**Keywords:** imaging biomarkers, multimodal imaging, neurodegenerative diseases, plasma biomarkers, positron emission tomography (PET), structural MRI

## Abstract

Neurodegenerative diseases (NDDs), including Alzheimer’s disease (AD), Parkinson’s disease (PD), frontotemporal dementia (FTD), and amyotrophic lateral sclerosis (ALS), represent a major global health burden. Imaging biomarkers have emerged as important tools for improving the diagnosis, monitoring, and biological characterization of neurodegenerative diseases. Structural MRI, diffusion tensor imaging (DTI), functional MRI (fMRI), positron emission tomography (PET), hybrid PET/MRI and molecular imaging have transformed our ability to investigate neurodegeneration *in vivo* non-invasively. This review highlights updated information on how each imaging modality offers a unique window into different disease pathophysiology including regional atrophy, amyloid-β, tau, dopaminergic terminal degeneration, synaptic density (SV2A), and neuroinflammation. We also focused on the translational and evidence supporting biomarkers, appropriate use criteria for amyloid and tau PET imaging, and standardized quantification methods such as the Centiloid scale. The growing role of multimodal fusion, where imaging is increasingly integrated with scalable fluid biomarkers to enable “blood-first” strategies where high-risk patients are selectively referred to advanced imaging, improving feasibility and equity. Despite tremendous progress, there are still issues with their standardization, sensitivity, specificity, and clinical translation. Moreover, the review emphasizes the frontiers of α-synuclein and glial state-specific PET ligands, advanced diffusion models, and dynamic connectivity analysis to support precision medicine and mechanism-based trial design for NDDs.

## Graphical Abstract

**Figure:**
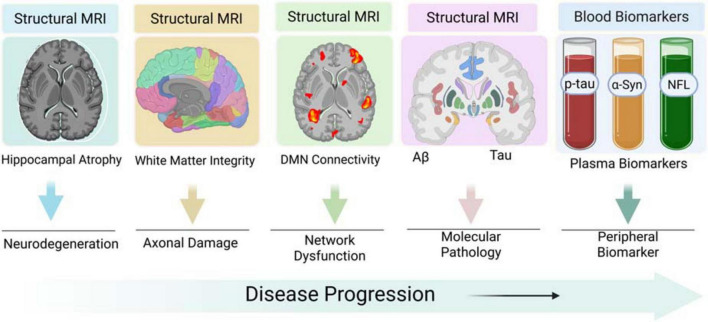
Multimodal biomarkers of neurodegenerative disease progression. Structural and functional neuroimaging markers, including hippocampal atrophy, white matter integrity, DMN connectivity, amyloid-β (Aβ), and tau pathology, together with blood-based biomarkers (p-tau, α-synuclein, and NFL), provide complementary insights into neurodegeneration, axonal damage, network dysfunction, and molecular pathology. These biomarkers facilitate early diagnosis, disease staging, and monitoring of disease progression.

## 1 Introduction

The neurodegenerative diseases (NDDs), including Alzheimer’s disease (AD) (Manzoor et al., 2021, 2022), Parkinson’s disease (PD) (Chakraborty et al., 2026; Taj et al., 2025), frontotemporal dementia (FTD) and amyotrophic lateral sclerosis (ALS) (Mullick et al., 2026) continue to pose an increasing burden globally. In 2025, an estimated 7.2 million Americans aged 65 or older are expected to live with AD, and this number is projected to double by 2060 in the absence of disease-modifying treatments (Alzheimer’s Association, 2015). Similarly, PD has been recognized as the world’s fastest-growing neurological disorder, and reportedly will affect nearly 12 million people globally by 2040 (Stocchi et al., 2024). Collectively, these NDDs lead to deteriorating mental and physical decline, which puts emotional and financial burdens on the patients, their caregivers and the healthcare system. Therefore, there is an urgent need for the identification of a reliable biomarker that can enable early diagnosis and effective therapeutic interventions.

The development of memory impairment in AD patients becomes evident years after undetected Aβ and tau protein accumulation in the brain (Gallardo and Holtzman, 2019). While PD motor symptoms arise after 50–60% of degeneration of the dopaminergic neurons (Surmeier et al., 2017). Non-invasive imaging biomarkers provide objective and quantitative measures of molecular, cellular and structural changes in the body (Moscoso et al., 2025), making them the promising candidate for diagnosis of NDDs. In recent times, the early detection of NDDs is largely made based on the defined pathophysiological hallmarks/framework. The 2018 National Institute on Aging–Alzheimer’s Association [AT(N)] research framework defined AD using biomarkers centered around amyloid (A), tau (T), and neurodegeneration (N), independent of clinical phenotype (Jack et al., 2018a). However, the 2024 revised updates created links between imaging technology and cerebrospinal fluid and blood-based biomarkers, which helped in timely evaluation of disease progression and clinical research cohort identification (Sperling et al., 2024). Following these advances, positron emission tomography (PET) introduced standardized acquisition and quantitative interpretation protocols which healthcare providers need to adopt to achieve clinical translation (Johnson et al., 2026). Further, centiloid scale (CL) allows to measure amyloid burden, by comparing results between different tracers and imaging sites for better clinical assessment (Klunk et al., 2015). This development reflects that the field of molecular neuroimaging has expanded rapidly for easy detection based on the amyloid pathology. The neuroimaging medical field has now been expanded further to the tau PET as its critical diagnostic tool for AD progression assessment and patient outcome prediction. Moreover, the development of second-generation tau tracers, including [18F]MK-6240 and flortaucipir, provide better specificity and superior signal-to-noise ratios in disease detection. Tau PET imaging has shown promise for assessing disease progression and improving biological characterization in AD and related tauopathies (Ossenkoppele et al., 2025; Tagai and Higuchi, 2025). Newer PET methods allow researchers to study the state of synaptic plasticity in the body. This *in vivo* measurement of synaptic density becomes possible through SV2A ligands, which include [11C]UCB-J and its derivative tracers (Carson et al., 2022; Estrada et al., 2016). Interestingly, the early development of cognitive decline observed in AD patients have shown a direct relationship with synaptic loss, which also exists in PD patients using the SV2A PET imaging (Martin et al., 2025; Ribarič, 2023). These findings suggest that ligand-based imaging approaches may provide valuable insights into early synaptic dysfunction in NDDs. Likewise, magnetic resonance imaging (MRI) further contributes additional diagnostic information through neuromelanin-sensitive imaging. MRI enhances the detection of substantia nigra neuronal degeneration in PD patients, and diffusion-based methods revealed the white matter pathology across NDDs (Amlien and Fjell, 2014; Shih et al., 2023; Welton et al., 2023).

Furthermore, neuroinflammation imaging has gained prominence in various aging and NDDs as microglial activation is increasingly recognized as a major contributor to neurodegeneration (Majumdar et al., 2026; Prajapati et al., 2025a; Prajapati et al., 2025b). PET tracers targeting the 18-kDa translocator protein (TSPO) allow *in vivo* assessment of neuroinflammatory processes. However, genetic polymorphisms and tracer limitations complicate the interpretation of TSPO-PET (Ramakrishnan et al., 2021). At a broader scale, initiatives such as the AD Neuroimaging Initiative (ADNI) and the PD progression Markers Initiative (PPMI) have standardized imaging pipelines by integrating imaging with fluid biomarkers and generated publicly available datasets to support biomarker validation and advanced analytics (Marek et al., 2025; Nosheny et al., 2024; Winberg and Persson, 2024).

Imaging biomarkers are now integral to therapeutic development and regulatory science. Amyloid and tau PET guide patient selection, monitor target engagement, and enable safety in AD trials, while dopaminergic imaging and neuromelanin MRI support clinical therapeutic decision-making in PD. Besides, the emergence of α-synuclein PET tracers and network-level imaging markers in FTD and ALS promise to transform into disease-modifying clinical trials. Therefore, this review elaborates on the collective advances in PET, MRI, and multimodal integration, highlighting how imaging biomarkers are emerging as a cornerstone of precision medicine for NDDs (Graphical Abstract).

## 2 Method

This narrative review is balanced and comprehensive by conducting a structured literature search on the topic. PubMed, Scopus and Web of Science have explored relevant publications containing the keywords including neurodegenerative diseases, imaging biomarkers, MRI, PET, DTI, fMRI, and other related keywords. The literature search focused on high-quality studies from the last 10–20 years that can contribute significantly to the field. Both preclinical and clinical studies that aim to translate imaging biomarkers for NDDs into clinical practice were included in the review. The studies included were selected for their methodologic strength, size, clinical importance, replicability, novelty, and impact within the domain of neuroimaging. The selection includes studies that examined the diagnostic capacity of neuroimaging techniques as well as studies that assessed disease progression. Other factors to be considered were studies on multimodal integration of neuroimaging and biomarkers and reviews and meta-analyses that provided important analysis. In that regard, because this article is not intended to be a systematic review but rather a narrative one, no PRISMA guidelines were utilized for screening, exclusion criteria, and quality assessment. However, an effort has been made to limit selection bias by selecting studies that covered several types of neuroimaging modalities, disease stages, and clinical presentations.

## 3 Imaging modalities and biomarker advances

Neuroimaging techniques provide critical insights to identify the basic pathological mechanisms underlying NDDs (Das et al., 2025; Prajapati et al., 2024). The most validated imaging biomarkers include structural magnetic resonance imaging (sMRI), diffusion tensor imaging (DTI), functional MRI (fMRI) and PET. Integration of multiple modalities such as structural, functional and molecular data using hybrid and multimodal approaches has become more common in medical imaging of NDDs (Jack et al., 2018a,b). Each modality provides unique advantages and constraints: sMRI detects large-scale tissue shrinkage patterns, DTI characterizes white matter microstructural integrity. fMRI maps the brain network connections, PET visualizes protein aggregation, synaptic breakdown, neuroinflammatory markers and hybrid PET/MRI enables the simultaneous acquisition of multiple imaging dimensions (Moscoso et al., 2022; Wijesinghe et al., 2025).

### 3.1 Structural magnetic resonance imaging (sMRI)

Structural magnetic resonance imaging (sMRI) remains the primary neuroimaging tool in both research and clinical practice, providing high-resolution anatomical brain images that quantify brain tissue volumes of gray matter, white matter and cerebrospinal fluid (Bigler, 2015). The most recognized sMRI biomarker for AD exists as hippocampal atrophy, which shows a direct relationship with cognitive deterioration and disease advancement (Jack et al., 2018a; Seiger et al., 2018).

**Preclinical evidence:** Preclinically, sMRI studies have validated multiple structural bioimaging biomarkers of NDDs through their capability to monitor brain deterioration that corroborated with the histopathological examinations (Abrol and Calhoun, 2025). Research using high-resolution MRI on transgenic mouse models of AD, including APP/PS1 and 3xTg-AD, has shown the progressive reduction of hippocampal and cortical volume (Chiquita et al., 2019; Delatour et al., 2006). Which closely mirrors the progression of amyloid accumulation, tau protein damage, synaptic deterioration and cognitive decline (Chiquita et al., 2019; Delatour et al., 2006). This research-based evidence demonstrates that hippocampal and medial temporal lobe atrophy serve as reliable biomarkers which connect the early pathological changes to measurable brain structural changes in human subjects. Similarly, quantitative susceptibility mapping (QSM) have helped in the detection of iron deposit-mediated nigrostriatal degeneration in the 6-OHDA and MPTP models of PD, thereby supporting its translational relevance (Virel et al., 2014). Studies have reported that interaction of iron with neuromelanin in the nigral region using the MRI techniques can form the basis of detection of neuromelanin in patients (Petiet, 2021). Nevertheless, research on animal models for FTD and ALS shows heterogenous findings. However, new preclinical imaging research demonstrates that structural MRI can track molecular changes in specific brain areas, which correspond to cortical and motor system deterioration observed in these diseases (Boxer et al., 2013).

**Clinical evidence:** sMRI remains the main neuroimaging tool in NDDs diagnosis because this method delivers high spatial resolution with higher reproducibility, enabling conduction of large multicenter studies (Huang and Zhang, 2023). The most well-established sMRI biomarkers in AD involve hippocampal and medial temporal lobe atrophy, both of which demonstrate direct relationships with memory deficits and disease progression and severity (Wolk et al., 2017). The Free Surfer and CAT12 pipelines enable researchers to perform volumetric and cortical thickness analysis for hippocampal subfields and temporoparietal cortical thinning across observational and therapeutic trials (Brown et al., 2020; Frisoni et al., 2010; Gaser et al., 2024). Moreover, the “brain age” models that utilizes the machine learning to analyze sMRI data, show that people with prodromal and asymptomatic amyloid-positive conditions experience faster brain aging relative to their cognitively impaired patients (Cumplido-Mayoral et al., 2024). In this context, the diagnostic capabilities of MRI have been enhanced for PD patients utilizing the structural imaging techniques that can detect early nigrostriatal degeneration. Additionally, the combination of Neuromelanin-sensitive MRI with 7T ultra-high-field strength allows to measure substantia nigra integrity with high accuracy. This is strongly proved to be beneficial for effective treatment strategies for PD as they help to timely understand the disease progression (Lakhani et al., 2024; Prasad et al., 2018). Besides, the iron-sensitive method QSM shows that iron deposits occur in the basal ganglia region and can be used to diagnose the severity of PD and atypical Parkinsonian syndrome (Ravanfar et al., 2021; Zhao et al., 2017).

Furthermore, the structural MRI has been proved to be beneficial in the diagnosis of FTD by visualizing the brain deterioration patterns using the frontal and anterior temporal atrophy. Similarly, the nonfluent variant PPA can be diagnosed from the left perisylvian–insular atrophy observed in sMRI (Lu et al., 2013; Rohrer et al., 2010). In ALS, the sMRI detects the degeneration of primary motor cortex and corticospinal pathways, the characteristic pathological phenomenon reported in the development and progression of the disease. Likewise, the combination of magnetization transfer imaging with cortical myelin mapping enables better detection of microstructural damage in the brain (Ghaderi et al., 2025; Verstraete et al., 2012).

In terms of a disease-based approach, sMRI proves highly applicable to Alzheimer’s dementia based on hippocampal and cortical atrophy assessments. It plays a major role in assessing degeneration of the substantia nigra in PD and atypical parkinsonian disorders. On the other hand, in frontotemporal dementia, specific regional patterns of atrophy are used for subtype determination; for ALS, structural MRI plays an important role in assessing motor system degeneration (Frisoni et al., 2010; Lakhani et al., 2024; Lu et al., 2013; Rohrer et al., 2010; Wolk et al., 2017).

Therefore, sMRI is widely used in clinical practice for the assessment of NDDs. However, it has limited ability to detect early molecular changes before overt degeneration occurs. As a result, it is best used in conjunction with other molecular/functional imaging techniques for improved detection of early diagnosis and disease staging.

### 3.2 Diffusion tensor imaging (DTI) and white matter integrity

Another MRI technique known as diffusion tensor imaging (DTI) uses white matter microstructure analysis to study neural tissue by studying water molecule diffusion patterns in different directions within the neuronal compartment. In healthy white matter, structures like axonal membranes, and myelin sheaths and cytoskeletal elements naturally restrict the water movement as these cellular compartments act as barriers to the process of diffusion. Therefore, DTI-derived metrics enable white matter integrity assessment by measuring these restrictions within the neuronal tissues. In this technique, the brain tissue reveals its directional fiber pattern through Fractional anisotropy (FA) measurements, which also demonstrate its structural arrangement. Further, the brain tissue reveals its complete diffusivity and its level of tissue empty space through mean diffusivity (MD) measurements. Similarly, the measurement of radial diffusivity (RD) shows demyelination, but axial diffusivity measures damage to axonal structures (Basser et al., 1994; Song et al., 2005). Hence, it can be said that the detection of DTI abnormalities occur before structural MRI can identify macroscopic atrophy, which makes DTI a valuable tool for identifying neurodegeneration in its earliest stages.

**Preclinical evidence:** Preclinical DTI research has been instrumental in validating the diffusion metric principles through its ability to connect imaging results with histopathological findings. The transgenic AD mouse models i.e., APP/PS1 and 3xTg-AD showed that FA decreases and MD increases using the DTI measurements (Mori et al., 2007; Wang et al., 2019). Further, in PD models, diffusion abnormalities occur in the nigrostriatal and corticostriatal tracts of 6-hydroxydopamine and MPTP models, which match the pattern of dopaminergic neuron destruction and axonal breakdown (Boska et al., 2007; Van Camp et al., 2009). The preclinical ALS models show reduced FA, which impact the corticospinal and motor pathways because these areas undergo axonal degeneration and motor neuron deterioration (Brunet et al., 2020; Zhang et al., 2018). Therefore, preclinical studies have shown that white matter tissue damage occurs because of diffusion abnormalities, which lead to microstructural changes in the tissue.

**Clinical evidence:** The DTI research-based white matter degeneration observed in NDDs have been consistently replicated in human studies. In AD, large-scale research has documented decrease in FA values decrease with elevated MD value. These diffusion pattern-based studies show that changes in these brain regions occur during the mild cognitive impairment stage and even in the amyloid-positive individuals who are not yet symptomatic (Acosta-Cabronero et al., 2012; Kantarci et al., 2017; Peter et al., 2025). Further, these diffusion changes also directly correlate with both amyloid and tau accumulation, and can help predict rate of the dementia advancement (Kantarci et al., 2017). Likewise, PD patients exhibited microstructural damage of their nigrostriatal pathway through DTI, evident from the decreased FA values specifically in the substantia nigra and putaminal connections (Langley et al., 2016; Zhang and Burock, 2020). This could help doctors in distinguishing typical Parkinson’s disease from atypical Parkinsonian syndromes. Notably, similar brain diffusion abnormalities have been reported in people with idiopathic REM sleep behavior disorder, which frequently develops into synucleinopathy (Rahayel et al., 2018). Behavioral variant frontotemporal dementia (FTD) is characterized by extensive white matter damage across frontotemporal association fibers, the uncinate fasciculus, and corpus callosum interhemispheric tracts. The FA measurements in these areas show a direct relationship with the severity of behavioral problems and social abilities deterioration (Mahoney et al., 2014). Moreover, ALS produces the most prominent DTI markers among all neurological degenerative diseases because it leads to substantial FA reductions, which affect the corticospinal tracts, internal capsule, and cerebral peduncles that correlate with motor function decline, disease progression, and even patient survival (Agosta et al., 2010; Schuster et al., 2024). The more advanced diffusion modeling frameworks such as NODDI and FBA add further precision in disease diagnosis because they allow researchers to measure axonal density independently from their fiber orientation assessments.

Overall, DTI has become an indispensable tool for mapping white matter changes across the full spectrum of NDDs. Its strengths lie in early detection, disease progression staging, and network-level insights when paired with connectomics. While limitations including susceptibility to motion and acquisition variability remain, growing methodological standardization and advanced modeling are enhancing these modeling approaches and their reliability for multicenter clinical trials.

DTI’s translational value in neurodegenerative disorders varies as well. In AD, DTI offers great significance in diagnosing subtle white matter lesions that precede the formation of cortical atrophy. While in PD, DTI mainly helps in identifying degeneration of the nigrostriatal tract. In FTD and ALS, DTI plays a vital role in understanding network disconnections and corticospinal tract abnormalities, respectively (Acosta-Cabronero et al., 2012; Kantarci et al., 2017; Langley et al., 2016; Zhang and Burock, 2020).

Despite the clinical potential of DTI, several limitations including methodological variability, susceptibility to motion artifacts, and the challenge of accurately interpreting complex tissue structures have impeded translation to the clinic. To improve the biological specificity of imaging strategies incorporating diffusion characteristics, advanced diffusion models, as well as multimodal imaging approaches, are required.

### 3.3 Functional MRI (fMRI)

Functional MRI (fMRI) works by detecting the changes in blood-oxygen-level–dependent (BOLD) signal fluctuations across the brain, which reflects neural activity through the neurovascular coupling process. Basically, the neuronal firing is reportedly proportional to the cerebral blood flow and oxygen consumption (Ogawa et al., 1990). The BOLD signal reveals the correlations between different brain regions during the resting-stating scans (rs-fMRI) that allow researchers to study brain networks. Similarly, during the task-based fMRI highlights the specific brain areas that get activated during cognitive, sensory, and motor tasks. Therefore, the detection of network and functional dysfunction in NDDs at their early stages becomes possible through fMRI.

**Preclinical evidence:** Research using preclinical fMRI has created a scientific framework to understand BOLD connectivity changes through its connection to synaptic dysfunction, neurotransmitter imbalances and circuit reorganization. Research using transgenic AD models has revealed through rs-fMRI that the brain connectivity between hippocampus, cortex and default-mode circuits becomes disrupted (Grandjean et al., 2014; Shah et al., 2013). Importantly, this hypoconnectivity reported during the rs-fMRI indicates genuine synaptic breakdown rather than outright neuron death. Further, research using fMRI has also proved beneficial in PD animal models that showed dopamine neuronal loss that is caused due to changes in brain connections between basal ganglia and cortex. These observed physiological changes mirror the motor deficits and irregular brain wave patterns recorded in direct brain recordings (Gao et al., 2017). Research using FTD and ALS preclinical models showed different trajectory in the frontotemporal and motor network degeneration patterns. In these NDDs, there were enhanced network connections, possibly as a compensatory response, before eventually collapsing as the disease advances (Trojsi et al., 2018). Collectively, the research conducted preclinically establishes that fMRI connectivity alterations in NDDs stem from actual changes which occur at the circuit level.

**Clinical evidence:** Scientists employ fMRI technology in clinical environments to study NDDs that cause brain network breakdowns. The default mode network (DMN) shows hypoconnectivity in AD according to rs-fMRI studies (Brier et al., 2012). Crucially, the disruption of DMN becomes detectable in individuals who have amyloid buildup but still cognitively normal. This serves as a predictor which helps doctors to identify individuals with mild cognitive impairments that will soon develop AD dementia (García-Colomo et al., 2024; Sperling, 2014). Similarly, research using fMRI with PD patients uncovered irregular neural connections between the basal ganglia, thalamus, and cortex that underline both motor disabilities as well as cognitive processing problems. Further, research conducted using this imaging technique reported that motor and cognitive deficits observed clinically occur due to changes in the putamen supplementary motor area and prefrontal cortex connectivity (Gao and Wu, 2016). Furthermore, research of last few years revealed that rs-fMRI connectivity patterns enable doctors to identify different PD clinical subtypes, including tremor-dominant and akinetic-rigid phenotypes (Chen et al., 2015). However, the network dysfunction in FTD operates through different mechanisms than AD because behavioral variant FTD (bvFTD) disrupts the salience network through damage to the anterior insula and anterior cingulate cortex (Seeley et al., 2007). However, svPPA causes anterior temporal network deterioration, which impacts semantic processing (Seeley et al., 2007). A recent study found that fMRI data obtained from FTD patients with tau-driven or TDP-43–driven subtypes presented different connectivity signatures, suggesting fMRI measurements show underlying molecular changes instead of just the clinical phenotypes (Brown et al., 2025).

ALS is also associated with marked functional network alterations. The first signs of ALS show hyperconnectivity between motor networks (Agosta et al., 2011, 2014). Research using graph theory to analyze fMRI data shows that NDDs cause network disruptions, which result in lower modularity, different hub arrangements, and reduced network operational efficiency (Bullmore and Sporns, 2009). Scientists now use newer emerging techniques to monitor brain network evolution over time, which enables them to identify disease progression and stages more effectively. Artificial intelligence has also brought major improvements to fMRI, which now generates better results for medical applications. The combination of deep learning with transformer-based models which use extensive datasets, leads to accurate predictions about how mild cognitive impairment will progress to AD (Latif et al., 2025). Together, these research findings demonstrate that fMRI functions as a powerful noninvasive method, which scientists use to study brain network disorders while developing personalized treatment approaches for NDDs. Although the indirect nature of the BOLD signal and numerous confounding physiological variables make it a tenuous biomarker. However, to evaluate truly disease-related network changes, fMRI is typically combined with structural and/or molecular imaging.

More importantly, the utility of fMRI in the context of each individual disease is dependent upon the underlying pathology, which involves changes in specific neural pathways and the disease’s characteristic phenotype. For AD, fMRI primarily demonstrates disruptions within the default mode network, which is linked to memory problems, while for PD, it highlights disruptions within motor and executive networks (Agosta et al., 2011, 2014; Brier et al., 2012; Brown et al., 2020; Gao and Wu, 2016).

### 3.4 Positron emission tomography (PET)

Scientists use PET-SPECT imaging to detect neuropathological changes through molecular imaging, which provides better results than structural or functional MRI. PET enables quantitative determination of protein aggregation, neurotransmitter function, synaptic density, and neuroinflammation measurements using radiolabeled ligands that bind to specific proteins, receptors, and enzymes. Therefore, PET serves as an essential tool for NDDs diagnosis, and patient classification and treatment evaluation.

**Preclinical evidence:** Preclinical PET studies function as an essential validation method which proves tracer specificity while establishing connections between PET imaging outcomes and biological disease processes. The AD models show that [11C]PiB PET tracers bind specifically to Aβ in the brain while showing direct relationships between PET results and the mouse brain plaque density and autopsy tissue analysis. This confirms that amyloid PET serves as a valid test for cerebral amyloidosis (Manook et al., 2012; Snellman et al., 2013). Further, the second-generation newer tau PET tracer i.e., [18F]MK-6240, demonstrates specific binding to paired helical filament tau in tauopathy models, which confirms their potential in human NDD diagnosis (Betthauser et al., 2019). Moreover, research on synaptic PET ligands which target SV2A protein has shown that PET signals match histological measurements of synaptic density for [11C]UCB-J and other similar compounds (Finnema et al., 2016). Research on neuroinflammation using TSPO PET tracers in animal models have demonstrated that the ability to detect active microglia and macrophages preclinically; however, the current tracers showed variable binding patterns, which requires developers to create newer tracers that are capable to identify and distinguish between different immune responses (Kreisl et al., 2020).

**Clinical evidence:** The medical field is largely dependent on amyloid PET as its main biomarker for diagnosing AD. The tracers [11C]PiB, [18F]florbetapir, and [18F]florbetaben successfully detect fibrillar Aβ deposits, which produce findings that align well with autopsy-based findings. A standardized measurement scale, the CL scale allows scientists to compare results across different tracers and testing sites. The scientific community uses CL values between 24 and 30 for both clinical and preclinical research purposes to detect positive amyloid deposits (Barthel et al., 2011; Rowe et al., 2017). The presence of amyloids on PET scans strongly indicates that patients with mild cognitive impairment will develop AD dementia, and this test is being used frequently by the doctors to select and stratify participants in clinical trials. Further, medical professionals now also use Tau PET for AD diagnosis, but scientists also use this technology to study tauopathy in different medical conditions. The tracers flortaucipir and [18F]MK-6240 correlate more closely with neurodegenerative and cognitive decline in AD patients. This enables better tracking of disease progression than amyloid PET scans.

In recent times, research has expanded beyond tau PET imaging to diagnose primary tauopathies, which include progressive supranuclear palsy (PSP) and corticobasal degeneration (CBD), through the identification of distinct binding patterns (Rowe et al., 2017). The development of newer technologies makes tau PET imaging an essential tool to diagnose patients and track their treatment responses. The imaging technique of PET allows researchers to visualize protein aggregation as well as synaptic loss and neuroinflammation, which represent the main causes of neurodegeneration. The synaptic PET imaging technique with [11C]UCB-J shows that AD, PD and FTD cause specific areas of synaptic density decrease, which strongly link to cognitive deterioration and provide better disease phenotype identification than structural atrophy measurements (Malpetti et al., 2023). The PET imaging technique of neuroinflammation detection through TSPO ligand [11C]PBR28 shows microglial activation in neurodegenerative diseases, yet researchers face challenges when interpreting results because of variation in TSPO’s binding affinity across individuals. The development of ongoing tracers which focus on astrocytic markers and microglial phenotypes works to solve these problems while enhancing the precision of biological measurements (Werry et al., 2019). For PD and related pathological conditions, dopaminergic imaging currently represents one of the most clinically established supportive imaging approaches. The neurodegenerative Parkinsonism diagnosis and tremor disorder differentiation and early disease detection become possible through the use of [123I]-FP-CIT SPECT (DaTscan) and [18F]-DOPA PET, which show presynaptic dopaminergic terminal loss (Quintas et al., 2024). PET and SPECT imaging provide highly informative molecular insights across NDDs, although clinical utility varies depending on tracer validation, disease context, and availability. Taking together, the growing collection of tracers spanning proteinopathies, synaptic integrity, neurotransmitter systems, and neuroimmune responses, makes molecular imaging the fundamental component for disease diagnosis and designing targeted clinical trials (Tables 1, 2).

**TABLE 1:** Comparative translational parameters and modality-specific considerations.

Modality	Sensitivity	Specificity	Accessibility	Cost	Clinical readiness
sMRI	Moderate	Moderate	High	Low–Moderate	Established
DTI	High (early WM changes)	Moderate	Moderate	Moderate	Emerging
fMRI	High (network dysfunction)	Low–Moderate	Moderate	Moderate	Research/Emerging
PET Amyloid/Tau	Very high	Very high	Limited	High	Advanced clinical
PET SV2A/TSPO	High	Moderate–high	Limited	High	Translational
Hybrid PET/MRI	Very high	Very high	Very limited	Very high	Specialized centers
Multimodal + fluid biomarkers	High	High	Increasing	Moderate	Rapidly emerging

**TABLE 2:** Imaging and multimodal biomarkers in neurodegenerative diseases.

Modality	Key Evidence / References	What It Measures	Disease Applications	Clinical Utility	Next Steps / Future Directions
Structural MRI (sMRI)	Delatour et al., 2006; Frisoni et al., 2010; Gaser et al., 2024	Gray matter atrophy, cortical thinning, iron load (QSM), neuromelanin loss	AD (hippocampal atrophy), PD (substantia nigra integrity), FTD (syndrome-specific atrophy), ALS (motor cortex thinning)	Diagnosis, progression tracking, trial endpoints	AI-based morphometry, ultra-high-field MRI, harmonized longitudinal metrics
Diffusion Tensor Imaging (DTI)	Agosta et al., 2010; Basser et al., 1994; Schuster et al., 2024	White matter microstructure, axonal integrity	AD (cingulum, callosal damage), PD (nigrostriatal tracts), FTD (frontotemporal tracts), ALS (corticospinal tract)	Early detection, network disconnection mapping	Fixel-based analysis, NODDI, multicenter harmonization
Functional MRI (fMRI)	Brier et al., 2012; Ogawa et al., 1990; Seeley et al., 2007	Network-level connectivity and activity	AD (DMN hypoconnectivity), PD (motor/cognitive circuit disruption), FTD (salience vs temporal networks), ALS (motor network plasticity)	Early functional biomarkers, subtype differentiation	Dynamic connectivity models, AI-based progression prediction
PET – Amyloid & Tau	Barthel et al., 2011; Klunk et al., 2015; Rowe et al., 2017; Tagai and Higuchi, 2025	Protein aggregation (Aβ, tau)	AD, PSP, CBD	Biological diagnosis, trial stratification	Earlier detection thresholds, wider tauopathy coverage
PET – Synaptic & Immune	Finnema et al., 2016; Kreisl et al., 2020	Synaptic density (SV2A), microglial activation (TSPO)	AD, PD, FTD	Disease severity tracking, mechanism-based endpoints	Astrocyte-specific tracers, microglial state specificity
PET/SPECT – Dopaminergic	Marek et al., 2025	Presynaptic dopaminergic function	PD, atypical parkinsonism	Diagnostic confirmation, prodromal enrichment	Integration with α-synuclein PET
Hybrid PET/MRI	Catana et al., 2012; Weiner, 2025	Structure + function + molecular pathology	AD, PD, FTD	Improved diagnostic accuracy, reduced patient burden	Routine clinical deployment, cost optimization
Multimodal + Fluid Biomarkers	Marek et al., 2025; Sperling et al., 2024	Imaging + plasma p-tau, NfL, α-syn	AD, PD	Scalable screening, precision stratification	Blood-first diagnostic workflows
AI-Enhanced Multimodal Imaging	Armanious et al., 2024; Ruan et al., 2024	Integrated pattern recognition across modalities	AD vs FTD vs VaD; MCI → AD	Prognosis, automated decision support	Federated learning, clinical validation, interpretability

The PET imaging applications depend on the molecular pathology of the disease. Amyloid and tau PET are currently the most advanced in AD, where they help diagnose the disease biologically, stage it, and treat it. Dopamine imaging using PET/SPECT is especially significant in Parkinson’s Disease (PD) and atypical parkinsonism. SV2A and neuroinflammatory PET imaging are gaining significance in FTLD and ALS due to their relevance in synaptic dysfunction and neuroinflammation (Barthel et al., 2011; Klunk et al., 2015; Malpetti et al., 2023; Rowe et al., 2017; Tagai and Higuchi, 2025).

Although PET tracers provide molecular images with unprecedented specificity, several challenges, including cost, accessibility, and tracer limitations such as off-target binding and genetic variability, limit the general applicability of PET imaging. An important alternative approach could be the use of plasma biomarkers amenable to large-scale analysis.

### 3.5 Multimodal imaging and hybrid approaches

The combination of multimodal and hybrid imaging methods enables researchers to study neurodegenerative diseases through multiple structural, functional and molecular biomarkers, which produce information that single imaging methods cannot detect. The hybrid imaging system PET/MRI allows researchers to obtain high-definition MRI images and PET molecular data at the same time, which results in better image alignment and less scan-to-scan variation and shorter patient examination time than performing tests separately (Catana et al., 2012). The combination of these methods generates vital information which scientists need to study neurodegenerative diseases because various neurological changes emerge during different stages of disease progression. Research conducted in preclinical and translational settings demonstrated the biological basis of multimodal integration through studies which showed molecular changes preceded tissue breakdown and network breakdown. Scientists who study Alzheimer’s disease through models have proven that amyloid PET imaging with MRI enables them to detect amyloid accumulation before hippocampal atrophy becomes visible because subsequent brain changes require multiple imaging approaches to monitor disease advancement (Jack et al., 2010). *In vivo* imaging with 18F-FDG-and 18F-Florbetaben-PET/MRI detects pathological changes in the brain of the commonly used 5XFAD mouse model of AD (Franke et al., 2020). The research findings show biological evidence which proves that doctors need to use multiple imaging techniques to identify medical conditions.

The diagnostic accuracy and disease stratification capabilities of PET/MRI and multimodal pipelines have been proven through clinical applications. The combination of amyloid PET with structural MRI metrics, which includes hippocampal volumetry and cortical thickness measurements, produces better results than using either test independently for identifying AD patients and their progression from mild cognitive impairment to dementia (Seo et al., 2017). Research studies which unite PET/MRI imaging methods reveal that tau PET signal patterns become more accurate for predicting brain degeneration and mental decline when scientists use MRI atrophy maps to analyze their results, which leads to better predictive results. Doctors now perform diagnoses through a new method which combines imaging technology with fluid biomarkers. The medical field now uses plasma biomarkers, including phosphorylated tau (p-tau217) and neurofilament light chain (NfL), as cost-effective screening methods, which should only use PET or MRI scans for patients who show high-risk indicators. The multimodal system in this tiered system shows effectiveness in clinical research because it reduces both treatment costs and patient intrusion without affecting diagnostic accuracy (Sperling et al., 2024). The screening process for Parkinson’s disease (PD) uses three different methods, which include plasma or CSF α-synuclein assays and dopaminergic imaging through DAT SPECT or [18F]-DOPA PET (Hauer et al., 2025). The research methods show how doctors can identify neurodegenerative disease development in people through multiple biomarkers, which enable early detection before brain damage becomes permanent.

## 4 Disease- and stage- specific integration of imaging biomarkers

While neuroimaging techniques are frequently treated as individual modalities, their clinical and translational utility is best understood in the context of individual neurodegenerative diseases and stages of pathology. AD is the most biomarker-mature neurodegenerative disease, with MRI useful for the measurement of hippocampal atrophy and, in some cases, cortical atrophy. amyloid PET (Aβ-PET) has the highest level of biomarker maturity, with a strong correlation between Aβ-PET signal levels and post-mortem neuropathological burden. Tau-PET also has high biomarker maturity, providing data that may allow for the pre-symptomatic diagnosis of AD (Jack et al., 2018a; Villemagne et al., 2013). A/T/N classification also takes into account MRI measures of atrophy. In PD, dopaminergic (e.g., DAT-SPECT/PET) imaging provides the highest level of diagnostic accuracy for early differentiation from atypical parkinsonian disorders, while MRI findings are supportive but less accurate (Postuma et al., 2015). FTD has the lowest level of biomarker maturity of neurodegenerative diseases and is typically characterized by frontotemporal atrophy on MRI. Emerging PET tracers enable assessment of FTD subtypes, but biomarker validation remains an active area of research (Bang et al., 2015). ALS is the least understood of the neurodegenerative diseases with respect to utility of imaging biomarkers. MRI and DTI can reveal corticospinal tract degeneration as well as alterations in brain function and structure in ALS (Brown and Al-Chalabi, 2017). However, standardization of clinical imaging in this disorder remains an evolving area of research. Therefore, incorporating knowledge of modality-specific findings within the context of each neurodegenerative disease and stage of pathology improves both diagnostic accuracy and translational relevance.

Most importantly, there is marked variability in the use of imaging biomarkers according to the stage of disease and clinical context. In preclinical and prodromal forms of AD, molecular imaging modalities such as amyloid PET, tau PET, and p-tau in plasma have better sensitivity compared to structural imaging (Jack et al., 2018a; Ossenkoppele et al., 2025; Rowe et al., 2017; Sperling et al., 2024). In more advanced stages of AD, structural MRI and DTI hold increasing significance (Acosta-Cabronero et al., 2012; Kantarci et al., 2017; Wolk et al., 2017). Similar to AD, PD biomarkers such as dopamine imaging using PET or SPECT imaging show more applicability in the early and prodromal stages of disease progression. On the other hand, MRI imaging techniques and changes in functional networks have the potential for being useful in the advanced stages of PD (Lakhani et al., 2024; Prasad et al., 2018). Multimodal imaging techniques that integrate several types of imaging techniques such as structural, diffusion, functional, and molecular imaging are more likely to become important in diseases like FTD and ALS due to their heterogeneity and overlapping clinical presentations (Mahoney et al., 2014; Trojsi et al., 2018).

## 5 Comparative evaluation of imaging modalities in neurodegenerative diseases

The high clinical utility of structural MRI is attributable to the features of reproducibility, accessibility, and validated indices of hippocampal atrophy and cortical thinning (Frisoni et al., 2010; Gaser et al., 2024). The superior sensitivity offered by DTI in early detection of changes in white matter microstructure is offset by low specificity, reproducibility issues stemming from motion artifacts, variations in scanners’ performance, fiber crossings, and acquisition-related factors (Acosta-Cabronero et al., 2012; Kantarci et al., 2017; Zhang and Burock, 2020). While fMRI allows for the identification of functional network dysregulation, in particular default mode, salience, and motor network disorders, its clinical application is constrained by physiological confounders and the indirect nature of assessment based on neurovascular coupling (Brier et al., 2012; Brown et al., 2025; Gao and Wu, 2016). The advantage of PET consists of its unmatched specificity in measuring levels of amyloid, tau, dopaminergic dysfunction, synaptic density, and neuroinflammation. However, its wide-scale use is hindered by the constraints of cost, exposure to radiation, availability of tracers, and nonspecific off-target binding (Barthel et al., 2011; Finnema et al., 2016; Kreisl et al., 2020; Malpetti et al., 2023). The use of hybrid PET/MRI and multimodality imaging enables the combination of structural, functional, and molecular features of pathology, yet remains more difficult and expensive compared to the single-modality approach (Catana et al., 2012; Seo et al., 2017; Sperling et al., 2024). On the whole, the findings suggest that there should be a staging process using accessible tools like MRI and fluid biomarkers for screening and using PET and hybrid imaging for validation of disease staging (Jack et al., 2018b; Sperling et al., 2024).

Notably, there is significant variability in the scalability and clinical validation of imaging biomarkers depending on the mode used. Structured MRI is currently the most scalable and validated modality due to its widespread use, reduced costs, and existing standardization methods (Frisoni et al., 2010; Gaser et al., 2024; Huang and Zhang, 2023). However, DTI and fMRI are more sensitive indicators of early dysfunctions within neural networks and microstructures. However, they may have some acquisition and motion challenges that impede their scalability and uniformity in multicenter settings (Acosta-Cabronero et al., 2012; Brier et al., 2012; Kantarci et al., 2017). PET imaging is considered highly specific biologically, especially for biomarker analysis of amyloid and tau pathologies; however, its wide adoption may be hindered by the unavailability of the necessary tracer, radiologic requirements, and high costs (Klunk et al., 2015; Kreisl et al., 2020; Rowe et al., 2017). Nevertheless, it is believed that PET/MRI hybrid techniques and AI-assisted multimodal frameworks will yield optimal diagnostic and prognostic accuracy. However, they will have to undergo more validation studies, standardize regulations, and reduce costs prior to full-scale clinical implementation (Catana et al., 2012; Latif et al., 2025; Seo et al., 2017). Thus, the available research data suggests implementing a stratified translational framework where mass-suitable screening tests like plasma assays and structural MRI are applied at first, whereas molecular imaging is employed solely for diagnostic purposes (Jack et al., 2018a; Sperling et al., 2024).

## 6 Limitations and challenges

Medical practice faces multiple barriers which prevent multimodal imaging biomarkers from becoming widely accepted in their current form. The main obstacle to achieving reproducibility and large-scale coordination and analysis pipelines which produce different results. Notably, the level of clinical development for imaging biomarkers is highly variable depending on the modality and the disease under study. The structural MRI is an extensively applied tool in everyday clinical practice to evaluate the atrophy pattern and rule out other diseases, while amyloid PET and tau PET have better substantiated roles in specific scenarios regarding the diagnosis and research of Alzheimer’s disease. Moreover, dopaminergic imaging with the use of DAT-SPECT and [18F]-DOPA PET has already proven its utility in aiding the diagnosis of Parkinsonian disorders. However, SV2A PET, TSPO PET, α-synuclein PET, diffusion model analysis, dynamic fMRI, and AI-driven multimodal imaging are still considered investigational technologies. Second, biological specificity exists at different levels throughout different medical imaging techniques. sMRI shows signs of neurodegenerative changes which occur after the disease onset, but it does not start the disease process. The measurement of diffusion in MRI becomes challenging because of two main factors, which include the presence of crossing fibers and the impact of partial volume effects. The signals obtained from fMRI represent neural activity indirectly, while these signals become affected by age-related changes, vascular and metabolic factors. The PET imaging technique offers precise molecular identification, yet its use remains restricted because it produces radiation exposure and costs too much, and only a few locations have access to it, and sometimes the tracer binds to the wrong sites or changes how genes affect tracer binding. Third, accessibility and equity issues remain substantial. The current availability of PET tracers and ultra-high-field MRI and hybrid PET/MRI systems exists only in specific centers, which creates problems for both widespread implementation and worldwide usage.

## 7 Summary

Neuroimaging technology advancements have revolutionized neurodegenerative disease research through their ability to study disease progression by monitoring structural, microstructural, functional and molecular changes in living patients. Structural MRI enables researchers to identify strong indicators which show brain tissue deterioration, while diffusion MRI shows the first signs of white matter separation, and functional MRI shows how brain networks function abnormally, and PET/SPECT imaging represents highly specific information of protein aggregation, neurotransmitter dysfunction, synaptic loss, and neuroinflammatory processes in neurodegenerative conditions. Scientists can monitor disease progression from beginning to end using PET/MRI hybrid systems, which operate with multimodal pipelines. The combination of imaging biomarkers with fluid-based measures, which include plasma p-tau and neurofilament light chain and α-synuclein assays, has sped up research progress because it allows researchers to perform affordable, large-scale screening tests which lead to specific imaging verification procedures. The current medical field depends on artificial intelligence and machine learning to generate high-dimensional multimodal data, which enhances diagnostic precision, and disease progression forecasting and biologically based patient classification methods. The current medical advancements indicate a new approach which moves away from traditional symptom-focused diagnosis toward precise neurodegenerative treatment based on disease mechanisms.

## 8 Future perspectives

Scientists need to combine their research activities between imaging methods, and biofluid testing and computational data analysis to progress neurodegenerative biomarker studies. The near future requires three essential elements for achieving reproducibility and clinical confidence through harmonized acquisition standards, multicenter validation studies and open benchmarking initiatives. Blood-first diagnostic workflows, which use plasma biomarkers to select patients for advanced imaging, are likely to become increasingly important components of scalable diagnostic workflows. The upcoming decade will bring fast progress in molecular imaging, which will extend beyond current amyloid and tau PET methods to include α-synuclein and astrocytic markers, microglial state-specific tracers and synaptic subtype imaging for better disease mechanisms and therapeutic target identification. The primary purpose of Hybrid PET/MRI platforms is to establish connections between molecular disease patterns and both structural and functional imaging data. The combination of multimodal imaging with fluid biomarkers and advanced analytics will create individual disease fingerprints, which will help doctors perform early interventions and develop new clinical trial methods and treatment plans for each patient.
